# Highly Integrated MEMS-ASIC Sensing System for Intracorporeal Physiological Condition Monitoring

**DOI:** 10.3390/s18010107

**Published:** 2018-01-02

**Authors:** Ning Xue, Chao Wang, Cunxiu Liu, Jianhai Sun

**Affiliations:** 1 State Key Laboratory of Transducer Technology, Institute of Electronics, Chinese Academy of Sciences, Beijing 100190, China; cxliu@mail.ie.ac.cn (C.L.); jhsun@mail.ie.ac.cn (J.S.); 2 School of Electronic, Electrical, and Communication Engineering, University of Chinese Academy of Sciences, Beijing 100190, China; 3 Department of Engineering Product Development, Singapore University of Technology and Design, Singapore 487372, Singapore

**Keywords:** Temperature sensor, oxygen sensor, pressure sensor, multiple sensors, MEMS, CMOS-compatible process, intracorporeal physiological condition monitoring

## Abstract

In this paper, a highly monolithic-integrated multi-modality sensor is proposed for intracorporeal monitoring. The single-chip sensor consists of a solid-state based temperature sensor, a capacitive based pressure sensor, and an electrochemical oxygen sensor with their respective interface application-specific integrated circuits (ASICs). The solid-state-based temperature sensor and the interface ASICs were first designed and fabricated based on a 0.18-μm 1.8-V CMOS (complementary metal-oxide-semiconductor) process. The oxygen sensor and pressure sensor were fabricated by the standard CMOS process and subsequent CMOS-compatible MEMS (micro-electromechanical systems) post-processing. The multi-sensor single chip was completely sealed by the nafion, parylene, and PDMS (polydimethylsiloxane) layers for biocompatibility study. The size of the compact sensor chip is only 3.65 mm × 1.65 mm × 0.72 mm. The functionality, stability, and sensitivity of the multi-functional sensor was tested ex vivo. Cytotoxicity assessment was performed to verify that the bio-compatibility of the device is conforming to the ISO 10993-5:2009 standards. The measured sensitivities of the sensors for the temperature, pressure, and oxygen concentration are 10.2 mV/°C, 5.58 mV/kPa, and 20 mV·L/mg, respectively. The measurement results show that the proposed multi-sensor single chip is suitable to sense the temperature, pressure, and oxygen concentration of human tissues for intracorporeal physiological condition monitoring.

## 1. Introduction

Recently, advanced microfabrication, including complementary metal-oxide-semiconductor (CMOS) and micro-electromechanical systems (MEMS) technologies, have been enabling the fast development of various modern biomedical sensors of higher accuracy, good reliability, and excellent sensitivity with lower cost, low power consumption, and small form factor [1,2]. Also, recent progress in wireless communication technology and ultra-low power integrated circuit technology have made it possible to build miniaturized and reliable biomedical sensor systems for emerging biomedical applications, including e-healthcare and u-healthcare [3,4,5,6,7,8,9]. In the past decade, various biomedical sensors and systems have been developed for portable, wearable, and implantable monitoring applications. Especially, multiple biomedical sensor monitoring is essential for many clinical applications that require reliable and biocompatible physiological monitoring [10,11,12,13]. Intracorporeal physiological condition monitoring such as pressure, oxygen, and temperature monitoring is widely used during the illness precaution and treatment [11,12,13]. Examples that require such multi-parameter monitoring are intracranial monitoring [14,15,16] and intra-abdominal condition monitoring [17,18]. 

The intracranial multi-modality monitoring system exists to measure intracranial pressure (ICP), brain tissue temperature, and brain oxygen concentration. In the United States, the incidence of severe head injuries is about 2 million people [19]. Intracranial monitoring can help manage and control intracranial pressure to prevent secondary brain injury for patients with severe traumatic brain injury. Intracranial pressure over 15 mmHg above ambient pressure is considered as raised ICP [20]. For patients that have intra-abdominal hypertension and abdominal compartment syndrome, intra-abdominal pressure and temperature monitoring methods were adopted [21]. This syndrome would be caused by diseases such as high-volume fluid resuscitation, lleus, hypothermia, and anemia [22]. As for intracranial monitoring application, the oxygen partial pressure, which can be calculated from oxygen concentration, is a key parameter for revealing cerebral oxygen metabolism, which further indicates the prognosis situation after brain trauma. Normal intracrianial oxygen partial pressure should be kept to 25–55 mmHg. The intra-abdominal sensors can be integrated into a catheter and inserted into the abdomen cavity. For a typical intracorporeal condition, the intracranial sensing range is 765–805 mmHg, the O_2_ partial pressure is 0–100 mmHg, and the temperature range is 20–50 °C [20].

Conventional equipment uses individual sensing probes for intracorporeal multi-parameter monitoring [23]. However, the large size of the drilling hole used for the insertion of the monitoring devices may induce body tissue inflammation. Furthermore, the fixture of the device on the body may cause the patient to become uncomfortable. The ease of probe dislocation can also lead to the electrical disconnection of the monitoring system. Therefore, a compact sensing system with multiple sensors integrated on a single monitoring probe would be attractive and is required for such intracorporeal physiological monitoring applications due to its small wound cut, ease of operation, low cost, and low power consumption. Moreover, further integration of an ultra-low-power wireless transceiver into the single-probe multi-sensor monitoring system would facilitate 24-h real-time monitoring. In this research, we focus on the highly monolithic integration of multiple sensors and interface circuits in a single chip using a standard CMOS process and a CMOS-compatible MEMS process. 

Individual sensing elements including the temperature sensor, the pressure sensor, and the oxygen sensor have been widely reported [24,25,26,27,28,29]. Depending on the working principle, the temperature sensor can be classified into three major types, i.e., the thermocouple sensor, the resistive temperature sensor, and the solid-state temperature sensor. The first two types of sensor need to have a patterning of extra layers such as nickel, platinum, and chrome as the sensing element. Most of them are not very compatible with the CMOS fabrication process, and the introduction of noble metal would increase the device cost. The solid-state temperature sensor utilizes the bipolar junction transistor (BJT) device (which can be fabricated by the CMOS process) to sense temperature variation by exploiting the device’s characteristic that temperature change results in the change of the PN junction voltage in a BJT device [24]. In the pressure sensing, the capacitive, resistive, and piezoelectric pressure sensors are dominant in the literature and market [25,26]. The resistive and piezoelectric sensors need to build extra layers on the CMOS device that complicate the fabrication processing. In contrast to the resistive and piezoelectric sensors, the capacitive-based pressure sensor can be directly formed by utilizing the existing metal layers of the CMOS process [27]. In the oxygen sensing, the electrochemical sensor is commonly used based on the oxidation and reduction reaction principle. Due to its relatively simple structure and effective sensing performance, the three-electrode configuration with a working electrode (WE), a reference electrode (RE), and a counter electrode (CE) is widely adopted for electrochemical sensor design and fabrication. In electrochemical oxygen sensing, the reference electrode is to standardize the background potential, while the working electrode and the counter electrode are to record the various currents through the applied voltage during O_2_ redox reaction [28,29]. 

In this study, we propose a compact multi-modality MEMS-ASIC sensing system for intracorporeal physiological monitoring applications. By exploiting the CMOS-compatible property of MEMS post-processing steps, a solid-state temperature sensor, a parallel metal plate capacitive sensor, a three-electrode electrochemical oxygen sensor, and their respective interface AISC circuits are designed and integrated on a single chip for the proposed single-probe multi-sensor monitoring system. The multi-modality sensing interface ASIC circuit and the solid-state-based temperature sensor was first designed and fabricated by a 6 metal layer CMOS process. The parallel metal plate capacitive pressure sensor and nafion-based three-electrode electrochemical oxygen sensor were subsequently post-processed on the same single chip. This paper mainly focuses on the sensor structure design and fabrication by the CMOS-compatible processing, the biocompatible packaging, and the systematic characterization of the multiple sensors in a single chip.

The rest of the paper is organized as follows: Section 2 describes the overview of the proposed monolithic-integrated multi-sensor single chip. In Section 3, the detailed design, simulation, and fabrication of the capacitive-based pressure sensor is presented. In Section 4, the device characterization and biocompatible assessment results of the multi-sensor single chip are given. Finally, the conclusions are drawn in Section 5.

## 2. Overall View of the Proposed Single-Chip Multi-Sensing System

**Figure 1:**
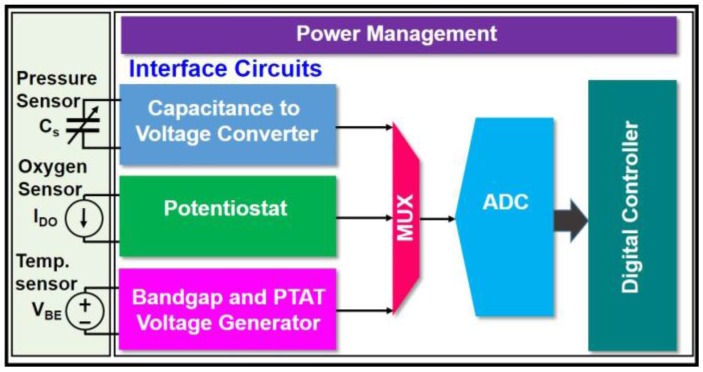
System block diagram of the single-chip multi-sensor consisting of pressure/oxygen/temperature sensors and integrated circuits.

Figure 1 illustrates the proposed highly monolithic-integrated multimodal sensing system consisting of a capacitive based pressure sensor, a three-electrode electrochemical oxygen sensor, a solid-state based temperature sensor, their respective sensor interface circuits, a readout multiplexer, an analog-to-digital converter (ADC), a digital control circuit, and a power management circuit. The interface circuit for the capacitive pressure sensor includes a pseudo-differential sensor bridge, a two-stage capacitance-to-voltage converter (CVC) to convert the capacitance changes into voltage output, and a self-calibration circuit to auto-calculate the baseline for process variation compensation. The oxygen sensor interface circuit has a negative feedback configuration operational amplifier as potentiostat and a transimpedance amplifier to convert the sensing current to output voltage. The solid-state temperature sensor has a bandgap reference and a proportional-to-absolute-temperature (PTAT) voltage generator to sense the temperature variation and convert it into an output voltage. A multiplexer is used to feed the three sensing signals to the 10-bit successive approximation (SAR) ADC to perform real-time data sampling and quantization in a time-multiplexed manner. The digital controller circuit provides the configuration and control for the rest circuits, performs digital filtering of the sensor signals, and also serves as a data interface to the external host. The power management circuit consists of low drop-out (LDO) voltage regulators to provide a 1.8 V voltage to supply the sensors and integrated circuits.

**Figure 2:**
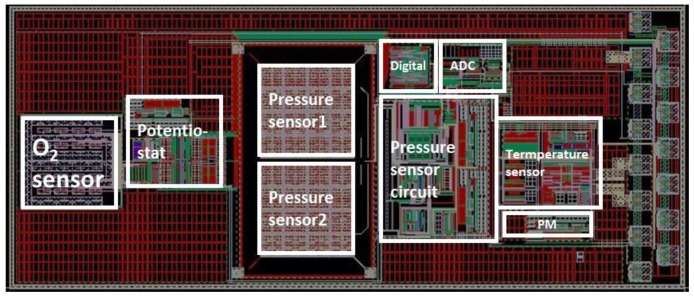
Layout of the proposed multi-sensor single-chip design.

Figure 2 presents the chip layout for the oxygen, pressure, and temperature sensors, sensor interface circuits, ADC circuit, digital control circuit, and power management circuit. The solid-state temperature sensor and integrated circuits were fabricated in a commercial 0.18-µm 1.8-V CMOS process. This standard CMOS process has 6 metal/dielectric layers for interconnection fabricated in the back-end of the line (BEOL) process. The structure of the capacitive pressure sensor including capacitive sensing metal layers, sacrificial metal layer for cavity releasing, and vias was also formed by the BEOL process of the CMOS in the commercial foundry. After the standard CMOS fabrication in the foundry, the wafers were sent back for in-house CMOS-compatible post-processing to complete the fabrication of the pressure sensor and oxygen sensor.

## 3. Pressure Sensor Design and Fabrication

### 3.1. Design and Simulation of the Pressure Sensor

The pressure sensor design includes the CMOS process in a 0.18-μm commercial foundry and MEMS post-process in our in-house fab. 

The MEMS structure of the pressure sensor is formed by fully utilizing the standard 0.18-µm CMOS process. In the CMOS process, there are six metal layers (M1-M6) with five 0.9-µm-thick SiO_2_ intermetal dielectric layers (IMD1-IMD5) in between each metal layer above the active transistor region. M1-M6 are made of aluminum with thicknesses of 0.54 µm (M1-M5) and 1.5 µm (M6), respectively. The passivation layers cover the M6 by the 1.35-µm-thick SiO_2_, 1.1-µm-thick tetraethyl-orthosilicate (TEOS), and 0.3-µm-thick SiN. Each metal layer can be connected by 0.26 µm × 0.26 µm via hole filled by aluminum. In the in-house MEMS process, the passivation layers and M6 are removed for post-processing.

**Figure 3:**
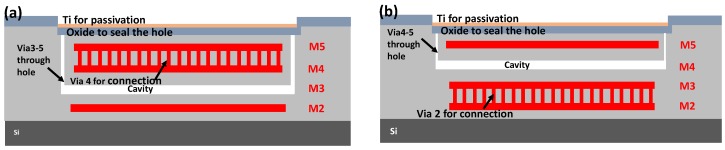
Pressure sensor design layout. (**a**) Design 1: M2 and M4/5 are the two plates of the capacitor; M3 is the cavity by etching away. (**b**) Design 2: M2/3 and M5 are the two plates of the capacitor; M4 is the cavity of the sensor.

The capacitive pressure sensor was fabricated in a metal-insulation-metal (MIM) sandwich structure. Two design layouts (Figure 2) were implemented and fabricated to evaluate their performance outcomes to find out the optimum pressure sensor structure with highest sensitivity while maintaining the high device yield, which are illustrated by Figure 3a,b, respectively. One metal layer (e.g., the M3 and M4, in Figure 3a,b, respectively) was removed to form the cavity. Thus, the structure above the cavity is the pressure sensitive membrane. 

In the Design 1 (Figure 3a), the M2 works as the bottom plate of the capacitive pressure sensor, while the M4/5 (connected through via hole) is the top plate. M3 was etched away through the connected via 3-5. The via hole was subsequently filled by SiO_2_ and Ti for vacuum sealing. Similarly, in the Design 2 (Figure 3b), the pressure-sensitive membrane is IMD4/M5/IMD5 and passivation layer, whereas M2/3 and M5 are the bottom and top plates of the capacitive pressure sensor. Table 1 lists the parameters of the two capacitive pressure sensor designs.

**Figure 4:**
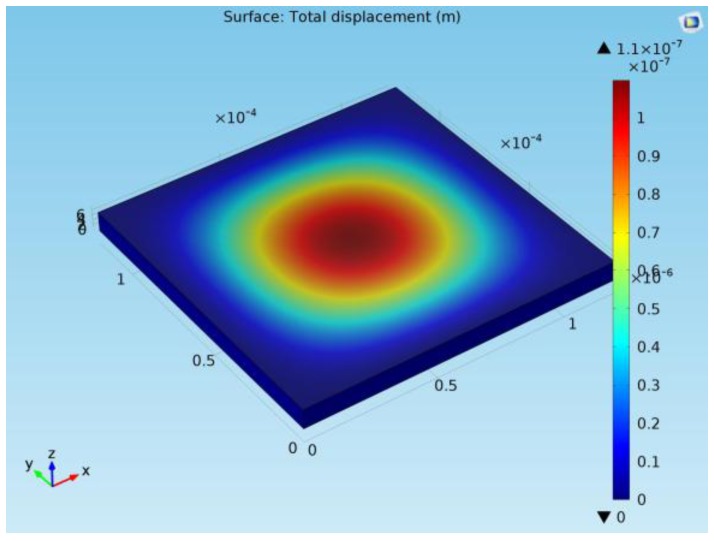
Simulation of the capacitive pressure sensor (120 µm × 120 µm) at 120 kPa.

The finite element method (FEM) software COMSOL was used to simulate the bending of the pressure sensors under applied pressures. The model was simplified as follows: The sealing holes were removed, and only two metal layers (top and bottom) were used for the simulation. The initial residual stress of the oxide was set to −100 MPa for both x and z directions. In the simulation, the mass density of Al and SiO_2_ are 2.7 × 10^3^ kg·m^−3^ and 2.2 × 10^3^ kg·m^−3^, respectively. The Young’s modulus of Al and SiO_2_ is 72 and 70 GPa, respectively. The Poison’s ratio of Al and SiO_2_ is 0.35 and 0.17, respectively. Electromechanics module in the COMSOL was used to achieve both the structure deformation and capacitance information under applied pressure at z direction. As an example, the simulation result image of a 120 µm × 120 µm capacitive pressure sensor design at 120 kPa is presented in Figure 4. 

**Figure 5:**
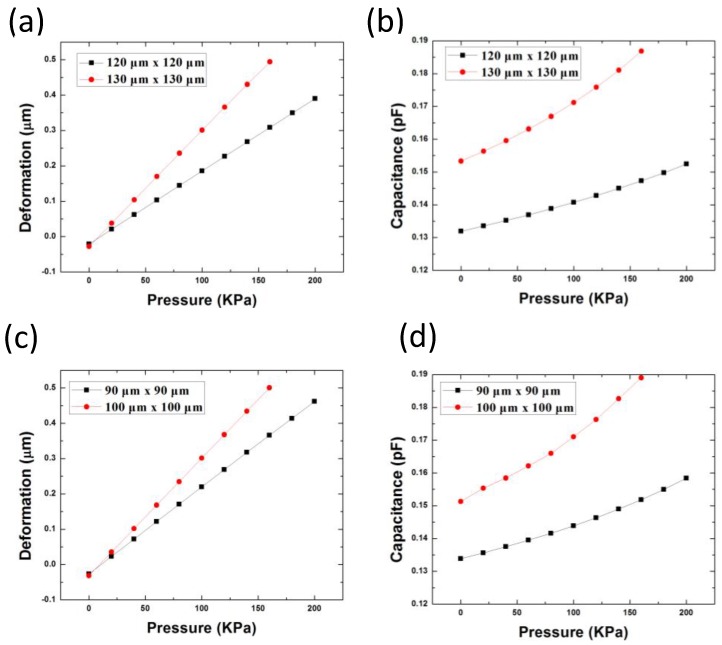
Simulation results for pressure sensor Design 1: (**a**) deformation; (**b**) capacitance, and Design 2; (**c**) deformation; and (**d**) capacitance with applied pressure.

The simulation results of the two capacitive-based pressure sensor designs with different sensor sizes are given in Figure 5. From the simulation of the Design-1 structure in the range of 0–200 kPa, the capacitance has relatively linear relationship with the pressure. The device sensitivity is 0.1016 af/Pa for device dimension of 120 µm × 120 µm, while the sensitivity is 0.2311 af/Pa for device dimension of 130 µm × 130 µm. In Design-2 structure, the device sensitivity is 0.112 af/Pa for device size of 90 µm × 90 µm, while the sensitivity is 0.241 af/Pa for device size of 100 µm × 100 µm. It is noted that the device sensitivity is similar for Design 1 with 120 µm × 120 µm and Design 2 with 90 µm × 90 µm. The sensitivity is also similar for Design 1 with 130 µm × 130 µm and Design 2 with 100 µm × 100 µm. At zero pressure, the membrane deformation has negative values due to the initial stress into the Al and SiO_2_. Thus, the membrane is initially bending outwards. At certain pressure, larger deformation induced from the larger membrane would result in touching of the bottom substrate. Thus, further increasing the membrane size would reduce the dynamic pressure sensing range. Moreover, it should be noted that larger device will cause the membrane collapse during the membrane releasing process. This failure mode will be discussed in the next section. Thus, it is critical to select the optimum sensor design structure and size.

### 3.2. Fabrication of the Pressure Sensor

**Figure 6:**
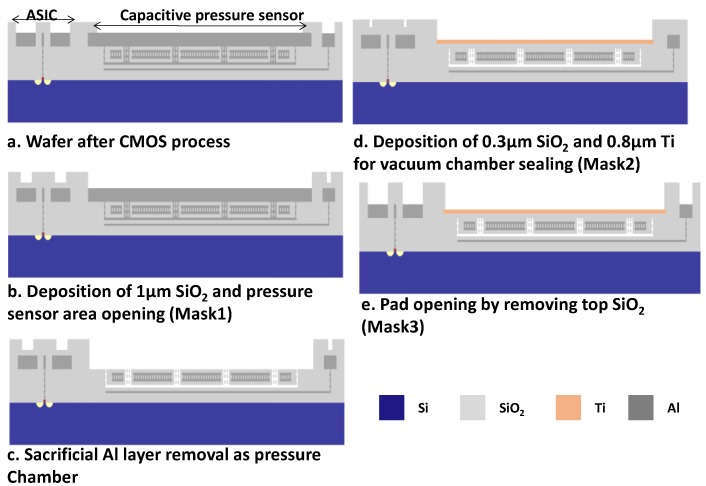
Schematic cross-section view of the post-processing micro-electromechanical systems (MEMS) process for pressure sensor.

The MEMS pressure sensor structure was formed in the CMOS fabrication, including the bottom electrode (M2 for Design 1, M2/3 for Design 2), middle metal for cavity (M3 for Design 1, M4 for Design 2), top electrode (M4/5 for Design 1, M5 for Design 2), and via connection from the middle metal to M6. Figure 6a is the structure after CMOS fabrication by the commercial foundry. Subsequent three mask sets were used to achieve the vacuum-sealed pressure sensor in the MEMS post-process. Firstly, 1-µm-thick PECVD SiO_2_ was deposited on the surface to protect the pad opening of the ASIC circuits in the silicon wafer. Next, the pressure sensor area was opened and the previous SiO_2_ layer was etched by reactive ion etch process (RIE) (Figure 6b). Then, M6, via, and middle metal were etched away by Piranha solution (H_2_SO_4_:H_2_O_2_ as 1:1). The wafer was then rinsed in distilled water (DI) and dried in CO_2_ supercritical dryer (Figure 6c). The cavity was opened in this process. The wafer was then coated by 0.3-µm thick low stress PECVD SiO_2_ and 0.8-µm thick titanium for vacuum chamber sealing. Titanium acts as high-density material for sealing, and, also, it can absorb moderate remaining gas in the chamber to keep the chamber at high vacuum level (<0.01 Torr). The Ti was etched outside of pressure sensor region (Figure 6d). Finally, the pads were opened by removal of the SiO_2_ (Figure 6e).

**Figure 7:**
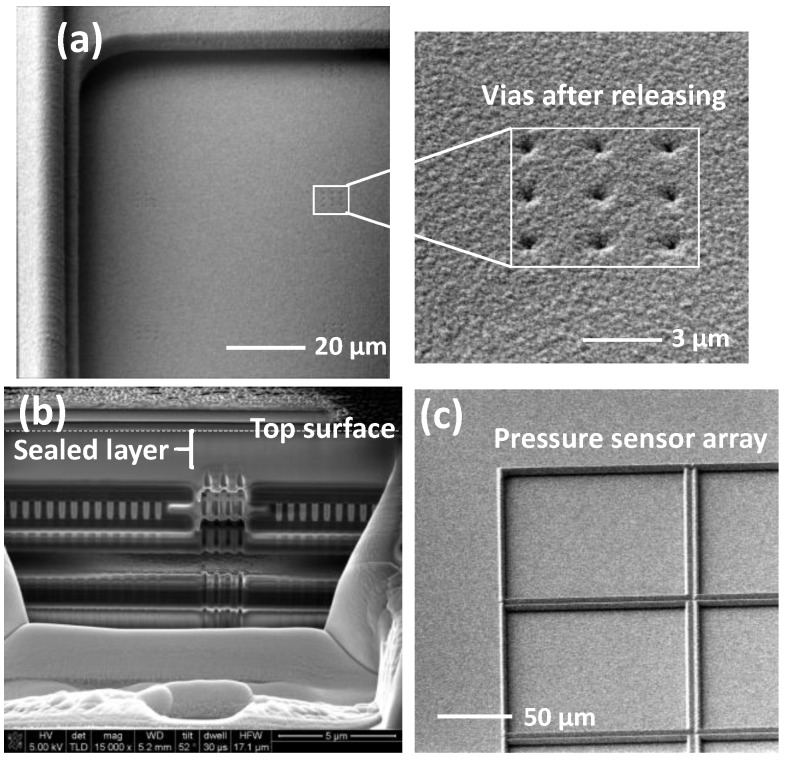
SEM image of the pressure sensor. (**a**) Opened via hole; (**b**) cross-section view of the etched via and cavity (M3); and (**c**) top view of the sealed via after SiO_2_/Ti vacuum sealing.

The key step in the post-process is the sacrificial metal layer release. The etching time and drying condition were finely tuned to ensure the middle metal layer, and vias are fully removed and the membrane is intact without collapse and obvious uneven surface. Figure 7a shows the scanning electron microscope (SEM) image at the completion of sacrificial layer and via removal. According to the 0.18-µm CMOS process, the via size is 0.26 µm × 0.26 µm. The 0.3-µm thick SiO_2_ and 0.8-µm thick Ti are sufficiently thick to deposit on the side-wall of via and seal the via5. After the sealing of via holes by SiO_2_ and Ti, the surface kept flat and holes were fully filled, as seen from Figure 7b,c. In Figure 7c, no sign of the via structure can be observed. In addition, from the cross-section view of the pressure sensor image in Figure 7b, the via was fully sealed with ~0.9 µm sealing layers on top.

**Figure 8:**
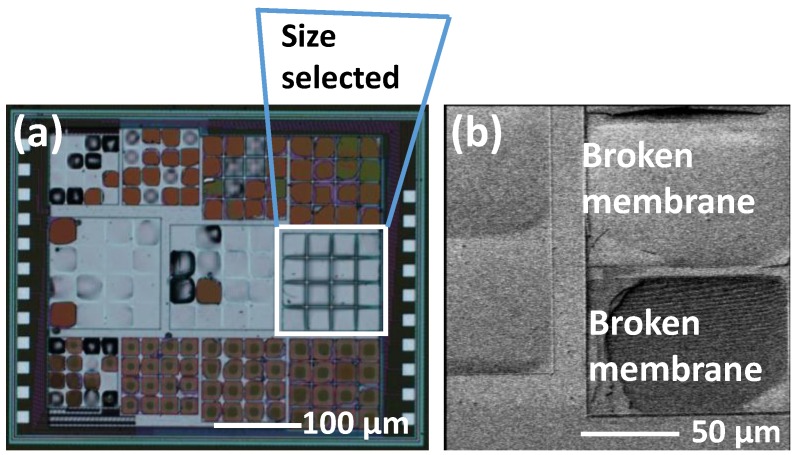
(**a**) Photo image of sensor array with different design and designs; (**b**) SEM image of damaged membrane.

To obtain the best performance of the sensor sensitivity during the design and fabrication of the MEMS-ASIC sensing system (Figure 2), we designed a test die (Figure 8) to find the optimum size of the capacitive pressure sensor. In the test die, only the pressure sensors were included without any control integrated circuit (IC). As mentioned above, there are two pressure sensor designs. In Figure 8, devices in row 1 and row 3 are the Design-2 type with thinner membrane (3.54-µm thick). In row1 or row 3, from column 1 to 3, the devices are the designs with sizes of 90 µm, 100 µm, 110 µm, and 120 µm, respectively. We can see that all the Design-2 type devices cannot be used due to the damage or collapse of the pressure sensor membrane. The membrane in Design-2 type devices are too thin to give mechanical support during wet etch process. Device in row 2 is the Design-1 configuration with sensor lengths of 140 µm, 130 µm, and 120 µm, respectively. Only the one with 120 µm × 120 µm has 100% yield sensor pixel. Therefore, we only chose the Design-1 device with 120 µm × 120 µm for our further device characterization. 

**Figure 9:**
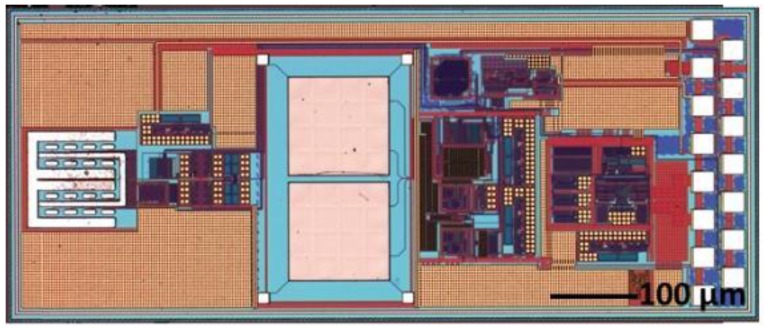
Photo image of the completed multi-sensor micro-electromechanical systems (MEMS)-application-specific integrated circuits (ASIC) chip.

In each sensor chip, we had 2 sets of 4 × 4 pressure sensor array to increase the overall sensitivity. The 16 sensors in each array were connected in parallel in the design. Thus, the total capacitance increased 16 times, decreasing the pressure detection limitation of the circuit. The differential sensor configuration of the pressure sensor array will reduce the noise floor. Figure 9 is the image of the integrated MEMS-ASIC system consisting of temperature-oxygen-pressure sensors and their interface circuits. Each component corresponds to the layout block in Figure 2. The bonding pads on the right side of the die were made of aluminum. Titanium was covered on top of the two differential pressure sensor arrays. 

### 3.3. Oxygen Sensor Design, Fabrication, and Packaging

**Figure 10:**
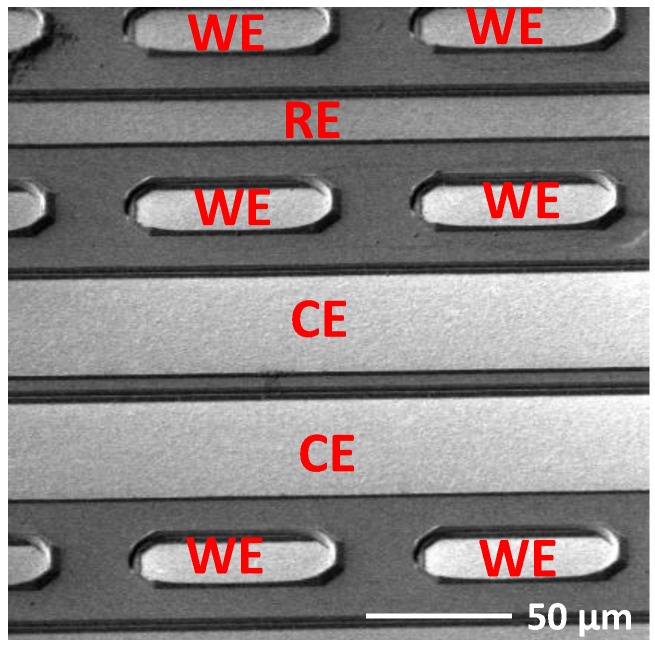
SEM image of the oxygen electrode.

A further photo-mask was used to pattern 200-nm thick platinum on the oxygen sensor electrode region for the working electrode (WE), reference electrode (RE), and counter electrode (CE). The 16 WEs have dimension of 50 µm × 25 µm. One RE and one CE are located at side of the WE (shown in the left region of Figure 9). The SEM image in Figure 10 gives a close look at the oxygen sensor electrode configuration.

A layer of biocompatible hydrogel-nafion (0.01 μL) was dip-coated on the oxygen sensor region. Nafion is a commonly used hydrogel to provide a solid-phase diffusion layer for oxygen transport [30]. Due to the inertial property of the platinum electrode, only O_2_ redox reaction occurs at the WE and CE as follows:

at WE side: O_2_ + 2H_2_O + 4e^−^ → 4OH^−^ (1)

at CE side: 4OH^−^ → O_2_ + 2H_2_O +4e^−^ (2)

The produced current is from hydroxide ions and is proportional to the oxygen concentration.

(3) $$I_{OH^{-}}=\frac{n\cdot A_{s}\cdot F\cdot D\cdot C_{DO}}{\delta }$$

where *n* is number of exchanged electron (*n* = 1), $A_{s}$ is the area of the WE, *F* is Faraday’s constant, D is the diffusivity of oxygen, $C_{DO}$ is the dissolved oxygen concentration, and δ is the thickness of the nafion layer. 

## 4. Device Characterization

**Figure 11:**
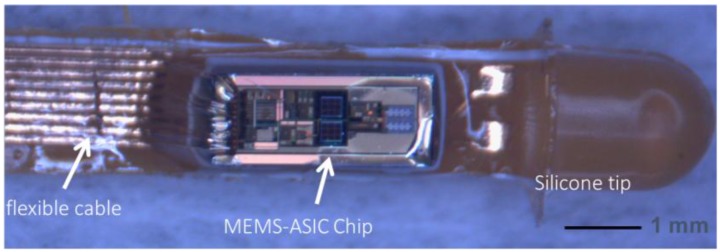
Biocompatible packaging of the multi-sensor MEMS-ASIC chip with flexible PCB.

Before the wafer dicing, 10-µm photoresist was spin-coated and the wafer was patterned. The regions of pressure sensor, oxygen sensor, and bonding pads were opened. Then, the wafers were diced into chips and cleaned by distilled water. The sensor chip was mounted into the flexible printed circuit board (PCB) cable and wire-bonded onto the bonding pads. 1-µm thick biocompatible parylene and ~50 µm thick PDMS (polydimethylsiloxane) were coated on the flexible PCB top surface. The remaining photoresist was lastly removed by acetone assisted by needle. The packaged image is shown in Figure 11. 

**Figure 12:**
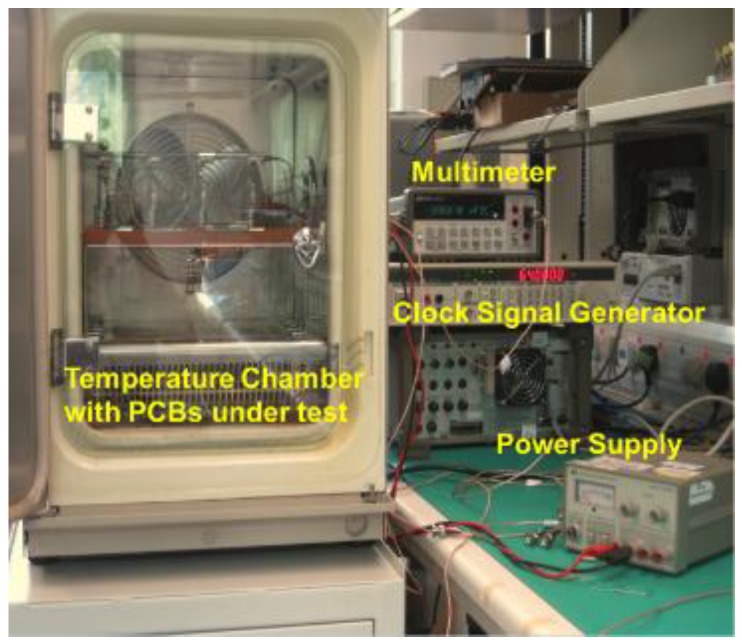
Characterization setup for temperature sensor calibration.

**Figure 13:**
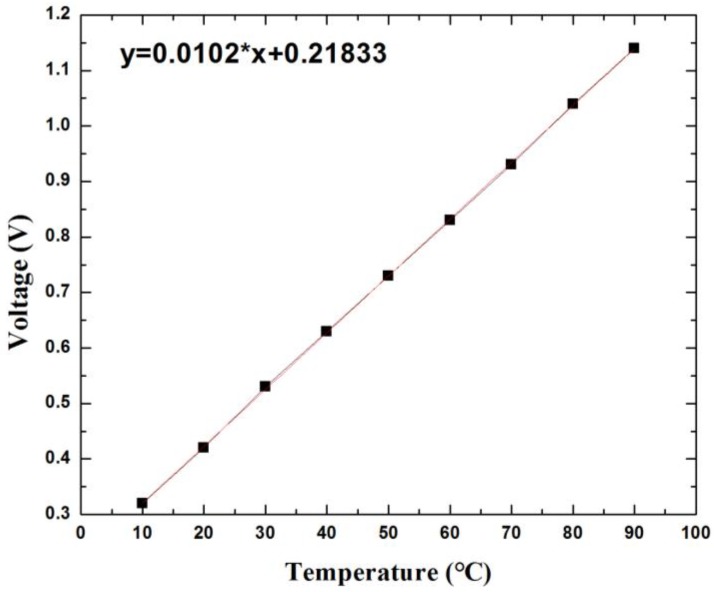
The calibration results of the temperature sensor.

For the temperature sensor calibration, the sensor was placed into a temperature chamber, and voltage and clock signals were applied to the sensor chip (Figure 12). The output voltage was recorded by a multimeter. From the result presented in Figure 13, the voltage response to the temperature variation is linear, with a sensitivity of 10.2 mV/°C.

**Figure 14:**
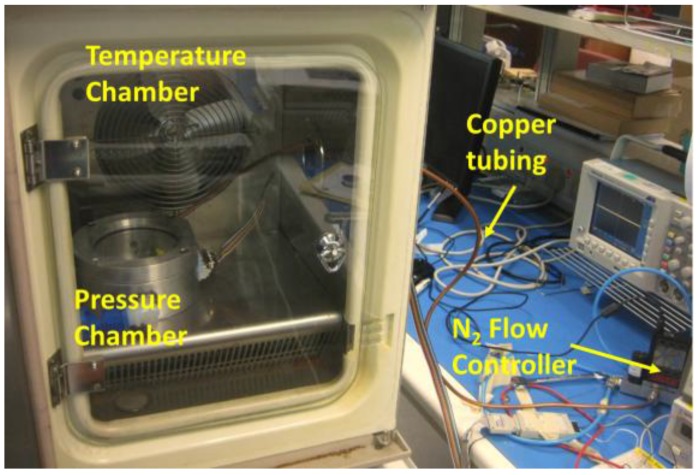
Characterization setup for pressure sensor calibration.

**Figure 15:**
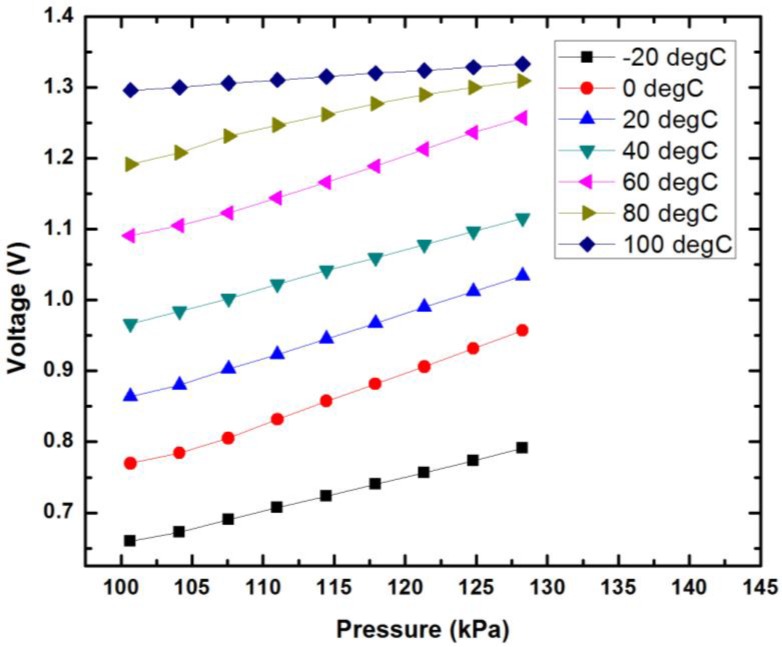
The calibration results of the pressure sensor at various temperatures.

In the process of the pressure sensor calibration, the chip was placed into a small pressure chamber with gas and pressure regulator connection. The small pressure chamber was then placed into a temperature oven for constant temperature control. Electrical cables were used to connect the sensor with the power supply and the clock signal generator. A multimeter was used for the output voltage measurement (Figure 14). Figure 15 shows the calibration results of the pressure sensor under temperature from −20 °C to 100 °C. The pressure sensor has similar sensitivity from −20°C to 80 °C at an average of 5.58 mV/kPa with a maximum 12% variation. In the application of physiological pressure monitoring, the pressure values are obtained from the calibration results with temperature compensation assistance from the solid-state temperature sensor. 

**Figure 16:**
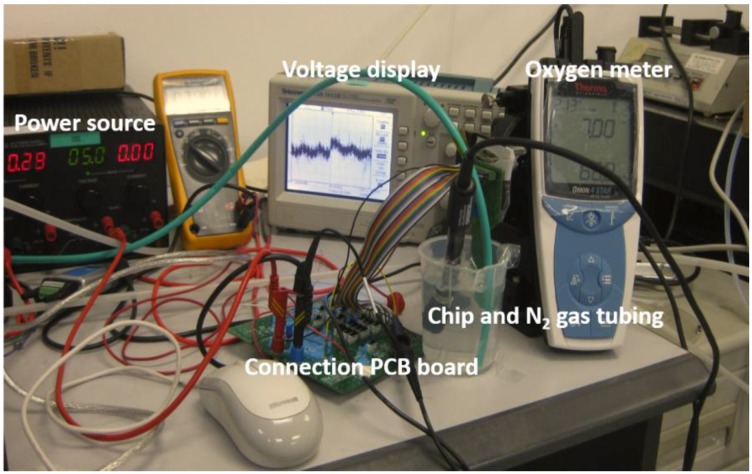
Characterization setup for oxygen sensor.

**Figure 17:**
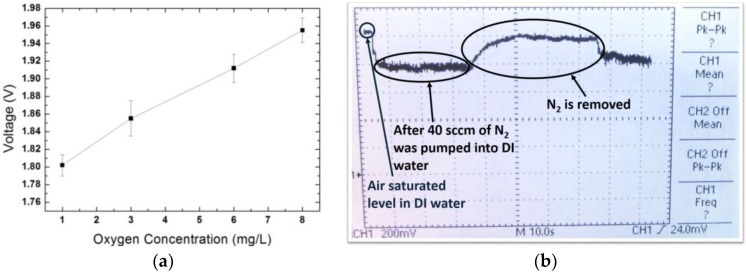
(**a**) Measurement results when water is filled with N_2_. (**b**) Transient response of the O_2_ concentration change.

The oxygen sensor setup was shown in Figure 16. The sensors were dipped into the DI water together with the commercial O_2_ meter. A N_2_ gas was pumped into the DI water to tune the dissolved oxygen concentration in the water. Figure 17a is the measurement result of the sensor response at different dissolved oxygen concentration. Figure 17b is the transient response of the sensor before and after pumping the nitrogen gas. The voltage output has high linearity and is stable at fixed oxygen concentration level. The sensitivity of the sensor is 20 mV·L/mg. The transient time after being filled with 40 sccm N_2_ gas from air saturated condition is ~3 s. The transient time after removal of the N_2_ gas to air saturated condition is ~15 s. The 15 s also includes the equivalent time that allows the air to dissolve in the water. 

Table 2 lists the concluded multi-modality sensing system performance and the physiological monitoring requirements of the three parameters (i.e., temperature, pressure, and oxygen concentration). The measured system sensing range fully covered the required range for the physiological monitoring and met the physiological monitoring accuracy requirement. For the stability measurement, the output variation after 30 days is 0.3%, 0.5%, and 5%, for the temperature, pressure, and oxygen sensors, respectively. The oxygen output shift is probably due to nafion degradation or delamination, which will be improved in our future research work. The corresponding measured sensing accuracy is ±0.2 °C, ±1 mmHg, and ±1 mmHg, respectively, which is suitable for our targeted physiological monitoring applications.

**Figure 18:**
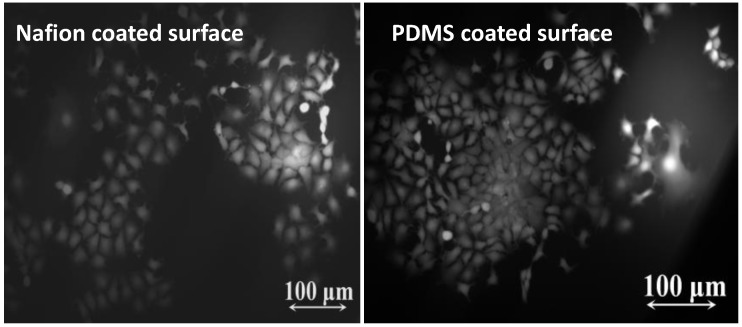
Cytotoxicity Assessment. **left**: Nafion coated surface, **right**: PDMS coated surface.

Cytotoxicity assessment was performed to verify the bio-compatibility of the device according to ISO 10993-5:2009 standards [31]. MCF-7 cells were cultured on the surface of the sensor with nafion coated surface and PDMS coated surface at 37 °C. At Day 3 of the assessment, the MCF-7 cells were totally flattened and spread in all directions to form a polygonal or spindle-line morphology (Figure 18). It is proven that the sensor sample has no cytotoxic effect.

Table 3 compares the proposed multi-modality sensing system with the state-of-the-art sensing designs. In contrast to other individual CMOS sensors, our proposed design integrated temperature sensor, pressure sensor, and oxygen sensor into a single chip using the CMOS-compatiable fabrication process. Thanks to the single-chip integration and biocompatible packaging, the proposed multi-modality sensing device enables single-probe monitoring, which results in benefits including small wound cut, ease of operation, and low cost, for intracranial and intra-abdominal monitoring. The sensitivity of our proposed multi-sensor is 10.2 mV/°C, 5.58 mV/kPa, and 20 mV·L/mg for temperature sensing, pressure sensing, and oxygen sensing, respectively. Due to the stringent size constraints imposed by the clinical requirement, our proposed design has smaller on-chip pressure and oxygen sensor dimension, and therefore obtains smaller sensor output signals.

## 5. Conclusions

A fully integrated MEMS-ASIC multi-sensor single-chip device consisting of a solid-state temperature sensor, a capacitive-based pressure sensor, and a three-electrode oxygen concentration sensor with integrated circuits has been designed, fabricated, and characterized. The fabrication of the multi-sensor device was done using a commercial standard CMOS fabrication and an in-house CMOS-compatible MEMS post-process. The device size is 3.65 mm × 1.65 mm × 0.72 mm. The multi-modality sensor chip was fully covered by biocompatible materials-nafion and PDMS. The cytotoxic test revealed that the device is cytocompatible. The characterization of the three sensors was also performed. All the three sensing functions have relatively linear responses and good sensitivities. The sensitivity of the sensors for the temperature, pressure, and oxygen concentration are 10.2 mV/°C, 5.58 mV/kPa, and 20 mV·L/mg, respectively. Therefore, the proposed multi-sensor single-chip device is suitable for intracorporeal physiological condition monitoring including intracranial and intra-abdominal monitoring. 

## Figures and Tables

**Table 1:** Design parameters of the pressure sensors.

	Sensor Dimension (µm)	Gap between Two Metals (µm)	Thickness of Pressure Membrane (µm)
Design 1	120 × 120 130 × 130	2.34 (0.54 µm cavity)	5
Design 2	90 × 90 100 × 100	2.34 (0.54 µm cavity)	3.54

**Table 2:** Multi-sensing system performance and requirement.

	Physiological Monitoring Requirement	Measured System Linear Sensing Range	Measured System Voltage Output Variation after 30 Days	Measured System Sensing Accuracy
Temperature	35–42 °C	10–90 °C	0.3%	±0.2 °C
Pressure (relative value)	0–60 mmHg (0–8 kPa)	0–187 mmHg (0–25 kPa)	0.5%	±1 mmHg
Oxygen partial pressure (Oxygen concentration)	22–100 mmHg (1–4.47 mg/L)	22–178 mmHg (1–8 mg/L)	5%	±1 mmHg

**Table 3:** Performance benchmark table.

	[24]	[27]	[30]	This Work
Process	0.16 μm CMOS	1.5 μm CMOS	MEMS	0.18 μm CMOS
Sensor type	Temperature sensor	Pressure sensor	Oxygen sensor	Pressure, temperature and oxygen multi-sensor
Sensing area	N. A.	0.2 mm^2^	0.43 mm^2^	0.6 mm^2^ (three sensors)
Sensitivity	N. A.	0.7 fF/mmHg 58.6 mV/kPa	633 pA/mmHg	10.2 mV/°C (Temperature) 5.58 mV/kPa (Pressure) 20 mV·L/mg (Oxgen)
Biocompatible packaging size	N. A.	6.4 mm (Diameter) 4 mm (Height)	1.1 mm × 1.5 mm (no IC)	3.65 mm × 1.65 mm × 0.72 mm
